# Student perceptions on enhancing the delivery of physical activity programs in a semi-rural university

**DOI:** 10.3389/fspor.2025.1627202

**Published:** 2025-10-01

**Authors:** Silindokuhle Sanele Radebe, Gerrit Jan Breukelman, Anné Suzanne Joubert, Lourens Millard

**Affiliations:** ^1^ Department of Human Movement Science, University of Zululand, KwaDlangezwa, South Africa; ^2^ Department of Nursing Science, University of Zululand, KwaDlangezwa, South Africa

**Keywords:** university students, physical activity participation, sport and recreation services, student perceptions, semi-rural South Africa, mixed-methods study

## Abstract

Adequate facilities and effective delivery of physical activity programs are essential for promoting active lifestyles among university students, especially in semi-rural contexts where structural barriers can hinder participation. This study investigated student perceptions and recommendations for enhancing the delivery of physical activity programs at the University of Zululand, a semi-rural South African institution. Using a mixed-methods design, quantitative data were collected from 328 full-time students via semi-structured questionnaires, while qualitative insights were drawn from 23 semi-structured interviews with purposively selected participants. Quantitative findings indicated moderate satisfaction with the marking and maintenance of sports facilities (*M* = 3.20, SD = 1.21, *p* = 0.004), but neutral perceptions regarding changing room conditions (*M* = 2.98, SD = 1.29, *p* = 0.787) and lighting (*M* = 2.76, SD = 1.24, *p* = 0.001). Perceptions of communication from the university's Sport and Recreation Services regarding plans and budgets were also low (*M* = 2.88, SD = 1.02, *p* = 0.036). Qualitative data supported these results, highlighting limited awareness of available programs, insufficient staff support, and a shortage of qualified coaches. Participants consistently recommended improved infrastructure, enhanced promotional strategies, greater recognition of student-athletes, and increased transparency in communication. These findings underscore the need for targeted institutional reforms to enhance visibility, accessibility, and support within university sport and recreation programs. The study offers practical guidance for institutional policy reform to strengthen the delivery of physical activity programs in semi-rural, under-resourced higher education institutions, ultimately fostering a more active, engaged, and healthier student population.

## 1 Introduction

Physical activity (PA) is widely acknowledged as a vital component of a healthy and balanced student lifestyle, contributing not only to physical well-being but also to academic success (1). Universities play a critical role in promoting student participation in physical activities by offering well-structured programs, accessible facilities, and clear communication about available opportunities (2).

However, research shows that many institutions, particularly those in semi-rural and historically disadvantaged contexts, struggle to deliver effective physical activity programs, with students frequently encountering service-related challenges such as limited access, outdated facilities, and inadequate institutional support (3). These problems are especially pronounced in historically disadvantaged universities (HDUs), where inequitable funding legacies rooted in apartheid-era higher education policies continue to undermine program delivery and participation (3–5). For example, Johannes et al. (3) found that under-resourced South African universities face systemic barriers including deteriorating facilities, weak communication structures, and limited student outreach. Similarly, Mthethwa (6), Nxumalo and Beetge (4) and Radebe et al. (7), reported that poor facility conditions, weak promotional efforts, and inequitable support deter student involvement in physical activity programs. These issues highlight the importance of contextualising student experiences within broader structural inequalities that shape higher education in South Africa.

Poor communication, low visibility of offerings, and insufficient promotion have also been repeatedly identified as major deterrents to participation in university physical activity programs (7). Nthangeni et al. (8), found that lack of awareness and inadequate institutional support were highlighted as critical barriers to female students' participation. Peters (9) similarly found that poor promotion and information gaps significantly reduced student engagement, underscoring the importance of visibility and communication. More recently, Kgokong and Parker (10) confirmed that student awareness, equitable access, and perceptions of institutional support remain decisive factors influencing participation. For the purpose of this study, the terms “sport” and “physical activity” are used interchangeably to reflect the range of both structured and informal programs provided through the institution.

While a growing body of literature has documented challenges to student participation in both structured and informal programs, relatively few studies have incorporated students' perspectives on how the delivery of physical activity programs can be enhanced. Although the importance of visibility, accessibility, and communication has been repeatedly emphasized (8, 10), few studies have directly explored students' perceptions on delivery of physical activity programs or gathered their recommendations for improvement (6). As such, this study aims to determine student perceptions on enhancing the delivery of physical activity programs in a semi-rural university. In doing so, it builds on existing research by confirming known limitations in the delivery of physical activity programs. It also extends the literature by providing student informed, actionable recommendations to improve visibility, communication, and overall effectiveness of these programs in semi-rural universities. This approach not only deepens our understanding of challenges in the delivery of physical activity programs but also provides practical insights for universities seeking to design more responsive and inclusive strategies.

## 2 Methods and materials

### 2.1 Study design

To determine student perceptions on enhancing the delivery of physical activity programs in a semi-rural university, this study employed a mixed-methods approach (explanatory sequential design). The research was divided into two phases, namely, a quantitative phase that involved the distribution of a structured questionnaire adapted from validated tools in previous literature (6), piloted with 20 students to ensure clarity. Internal consistency was confirmed using Cronbach's alpha, with scores of 0.895 (communication) and 0.771 (facilities) and a qualitative phase that involved semi-structured interviews with main questions adopted from previous studies (5, 6). This method facilitated the incorporation of both statistical analysis and in-depth examinations of student experiences and recommendations.

### 2.2 Participants and sampling

A total of 414 full-time students were recruited using a stratified random sampling technique to ensure representation across gender, academic level, and faculty. The sample size was calculated using the Raosoft online calculator (11) with a 95% confidence level, 5% margin of error, and an anticipated response distribution of 70%, yielding a minimum target of 318 responses. An additional 96 students were invited to account for non-response. Due to limited access to complete enrolment data from the university administration, stratification was estimated based on gender, level of study, and faculty using publicly available enrolment figures from the university website. All academic faculties at the institution were sampled as follows: Humanities and Social Sciences (*n* = 112), Science and Agriculture (*n* = 104), Education (*n* = 102), and Commerce, Administration, and Law (*n* = 96). The final sample included students aged 18–35 years: 176 identified as female, 149 as male, and 1 as other. All participants were enrolled in full-time programs at the KwaDlangezwa campus of the University of Zululand.

### 2.3 Data collection

Data collection commenced following ethical clearance from the University of Zululand Research Ethics Committee. Due to limited access to the central student registry, a multi-channel recruitment strategy was implemented in collaboration with academic staff, including Heads of Departments, lecturers, mentors, and sport and recreation personnel. Announcements were made in class and tutorial settings, supported by posters and emails. An information sheet outlined the study's purpose, procedures, and confidentiality assurances. Students returned signed consent forms along with their completed questionnaires, which were available in both the English and isiZulu language to accommodate preferences. Questionnaires were distributed in lecture halls, training venues, and tutorials, with a short completion time of 5–10 min.

Following the survey phase, a purposive sample of students was invited for follow-up interviews. Eligible participants were those who had completed the questionnaire, those involved in student leadership or sport-related governance structures (e.g., sports committees or recreation sub-councils), and were regularly participating in university physical activity programs. This selection ensured that interviewees had direct exposure to the delivery of physical activity programs.

Semi-structured interviews were conducted in English after obtaining written consent. Each interview lasted 15–20 min and focused on participants' assessments of current program delivery and their recommendations for improvement. Interviews were conducted over a two-week period. Additionally, the lead researcher conducted field observations of recreational facilities and operations, documenting impressions in a reflective journal to complement interview data.

### 2.4 Data analysis

Quantitative data were analysed using IBM SPSS Statistics for Windows, Version 25.0 (IBM Corp., Armonk, NY, USA), as guided by Pallant (12). Descriptive statistics (means, standard deviations) and *t*-tests (13) assessed levels of agreement with Likert-scale items about service quality, communication, and facility provision. These items were tested against a neutral midpoint value (3.0) to determine whether student perceptions were significantly positive or negative. A significance level of *p* < 0.05 was adopted. Exploratory factor analysis (EFA) was employed to identify underlying perception domains (14), using principal axis factoring with Promax rotation. Suitability for EFA was confirmed by a KMO value of 0.869 and significant Bartlett's test (*p* < 0.001). Two main factors emerged, communication effectiveness and facility conditions with cross-loading items removed to enhance construct clarity.

For the qualitative component, the researcher applied Lincoln and Guba's criteria of credibility, transferability, dependability, and confirmability to enhance trustworthiness (15). Prolonged campus engagement, a reflective journal, and methodological triangulation (survey results informing interview questions) supported credibility. Coding was conducted both deductively, from survey results, and inductively, from interview data, first manually using Erlingsson and Brysiewicz's framework (16), and then verified using NVivo 14 (17). Dependability was addressed through detailed analytic documentation and peer debriefing, while confirmability was supported through member checking with selected participants.

### 2.5 Ethical considerations

Ethical approval for this study was granted by the University of Zululand Research Ethics Committee (UZ-REC 0691-008). Participation was voluntary, with all students providing written informed consent. Confidentiality was maintained by assigning unique identifiers and excluding names from the dataset. Participants were informed of their right to withdraw at any stage without consequence.

## 3 Results

### 3.1 Quantitative results

To assess the level of agreement or disagreement with respect to their perception of the current university's physical activity programs service delivery, a one-sample *t*-test was used. The average agreement score for each perception was compared against a neutral score of 3. A mean score significantly higher than 3 (*p* < 0.05) indicates strong agreement with the perception, while a score significantly lower than 3 signifies strong disagreement. In cases where the result was not significant (*p* ≥ 0.05), it suggests that there is neither significant agreement nor disagreement regarding that specific perception. The findings, as summarized in Table 1, reveal that students generally agreed that the university provides clearly marked (*M* = 3.48, SD = 1.17, *p* < 0.001) and well-maintained sport facilities (M = 3.20, SD = 1.21, *p* = 0.004). However, they expressed disagreement regarding the adequacy of lighting (*M* = 2.76, SD = 1.24 *p* = 0.001), and were neutral about the cleanliness of changing rooms (*M* = 2.98, SD = 1.29, *p* = 0.787). In terms of communication by the institution, students agreed, that they had been informed about the benefits of physical activity (*M* = 3.42, SD = 1.41 *p* < 0.001), but expressed no significant consensus on other aspects such as departmental responsibilities, budget disclosures, or future service delivery plans.

**Table 1:** One-sample *t*-test of student perceptions of sport and recreation services.

My institution has.	*n*	Mean	Standard deviation	*t*	*df*	*p*-value	Direction
Clearly marked grounds and sport facilities	292	3.48	1.168	6.966	291	<.001*	Agreement
Sport grounds facilities that are well maintained	295	3.20	1.212	2.883	294	.004*	Agreement
Clean and well-maintained change rooms	296	2.98	1.289	−0.271	295	.787	Neutral
good lighting on facilities	296	2.76	1.243	−3.274	295	.001*	Disagreement
Informed me about the benefits of participation in physical activities	298	3.42	1.141	6.298	297	<.001*	Agreement
Communicated the responsibilities of Sport Administration Department official	296	3.03	1.062	.493	295	.623	Neutral
Communicated the UNIZULU’s Sport and Recreation budget for coming year	297	2.88	1.017	−2.110	296	.036*	Disagreement
Communicated the achievements of the Sport Admin Department	295	3.01	1.050	.111	294	.912	Neutral
Communicated the challenges faced by the Sport Admin Department in delivering sport services	297	2.90	1.024	−1.644	296	.101	Neutral
Communicated plans to improve service delivery in the coming year	298	2.94	1.087	−0.959	297	.338	Neutral

Direction = Agreement (*M* > 3, sig.), Disagreement (*M* < 3, sig.), or Neutral (*M* ≈ 3, ns); *n*, number of respondents; *M*, mean; SD, standard deviation; *t*, *t*-value; *df*, degrees of freedom; *p*, probability value.

* Indicates statistical significance (*p* ≤ 0.05).

Exploratory Factor Analysis (EFA) revealed two dominant constructs: The Communication factor (Cronbach's alpha = 0.895) which explained 47.5% of the variance and included items related to achievements, challenges, budgets, and service plans. In turn, the Facilities factor (Cronbach's alpha = 0.771) which explained 11.9% of the variance and encompassed perceptions of facility maintenance, signage, and cleanliness. A subsequent one-sample *t*-test showed a significantly positive mean score for Facilities (*M* = 3.23, *p* < 0.001), suggesting general satisfaction with infrastructure. In contrast, the Communication factor score (*M* = 3.03, *p* = 0.572) was not statistically different from neutral, indicating inconsistent or insufficient communication from the Sport Administration Department. Complete tables including item factor loadings (Supplementary Table S1), variance (Supplementary Table S2), and one-sample *t*-test results for each factor (Supplementary Table S3) are provided in the Supplementary Materials.

### 3.2 Qualitative findings

A total of 23 semi-structured interviews were conducted with students actively enrolled in physical activity programmes offered by the university's Sport and Recreation Department. These participants were purposively selected based on their engagement in sport, and many held leadership roles within student sport structures. The age and gender distribution of the interviewees is shown in Table 2, indicating that of the 23 participants, 15 were male (65.2%) and 8 were female (34.8%). Nearly half of the participants (47.8%) were aged between 23 and 26 years. The sample was also notable for its high level of student sport leadership, of which 13 participants (56.5%) had previously held or were currently holding positions on sport executive committees. Furthermore, the majority (65.2%) had been involved in the university's physical activity programmes for more than two years, giving them long-term experience with the institution's service delivery.

**Table 2:** Age and gender distribution of student-athlete interviewees.

Age group	Male	Female
18–22 years	2	3
23–26 years	7	4
27–30 years	5	1
Over 31 years	1	0

The analysis of the semi-structured interviews not only corroborated concerns highlighted in the quantitative findings but also provided a more nuanced understanding of how students perceive current sport and recreation service delivery, while giving them the opportunity to offer concrete recommendations for improvement. The objective of the interviews was to gain deeper insight into students' perceptions of how sport and recreation services are delivered at the university and to collect student-led suggestions for enhancing service effectiveness. By engaging students who were actively involved in sport and/or held leadership roles in sport-related committees, the qualitative phase complemented the survey findings by capturing first-hand experiences with institutional processes, communication practices, and support structures from the user's perspective. These emergent themes highlight both structural and perceptual gaps that require targeted attention from the university's Sport and Recreation Services.

#### 3.2.1 Promotional communication

Students repeatedly highlighted a lack of awareness regarding available sports programmes, especially among first-year students. Many only discovered sport offerings later in their academic careers:

“’m actually not sure how many sport codes are available.” (Que262)

“When I came here, I didn’t even know there was netball, I found out a year later.” (Que322)

“Most first years don’t know about the sports codes offered at the university.” (Que001, Que269, Que322, Que325, Que327, Que328)

This limited visibility discouraged early participation. Participants recommended better orientation sessions, frequent promotional campaigns, and use of social media to increase awareness and engagement:

“More events and recruitment drives would help students become more familiar with physical activity programs.” (Que317)

Additionally, students expressed a lack of understanding about the benefits of participation, suggesting communication strategies should also emphasise health and social outcomes:

“Many students don’t understand the benefits of participating in physical activity, and as a result, we lose serious talent.” (Que262)

#### 3.2.2 Student-athlete support

Participants cited unequal treatment across sports codes and gender disparities in access to equipment and support:

“Some sports, like tennis, get better maintenance, but other sports are treated unequally.” (Que264)

“Women’s sports don’t get the same recognition or support as men’s sports.” (Que321)

A lack of staff engagement was also reported, with students perceiving the sport department as largely absent:

“It would be motivating to see staff attend games. It feels like they just don’t care.” (Que286)

Administrative policies, such as the requirement to sign indemnity forms before travel, were seen as signs of inadequate institutional backing:

“*Whenever we participate in outdoor games, they require us to sign indemnity forms*.” (Que321)

Students suggested improved financial and motivational support including bursaries, discounted gym memberships, and recognition programs to foster participation:

“Awards or certificates would help us feel appreciated. That would motivate us to keep going.” (Que269)

#### 3.2.3 Infrastructure, equipment, and resources

Poor facility maintenance and lack of equipment emerged as another significant theme. Participants criticised inadequate drainage, safety hazards, and substandard changing rooms:

“They need to maintain the facilities more. The drainage system on the fields is terrible.” (Que269)

“The changing rooms are terrible, and most sport codes don’t even have changing rooms.” (Que319)

Equipment shortages were also noted, with delays in receiving basic items like balls and bibs:

“We’ve been asking for balls and bibs since the beginning of the year, and it’s already August.” (Que321)

The absence of essentials such as first aid kits and meals for away games further contributed to safety concerns and low morale:

“Some sports don’t even have first aid kits, yet the department expects students to give their all.” (Que322)

“There’ve been situations where food for a game was not provided.” (Que001)

Overall, students perceived a misalignment between institutional priorities and their needs. They advocated for more transparent, equitable resource allocation and improved service delivery to support meaningful participation.

## 4 Discussion

This mixed-methods study explores how university students perceive and experience the current delivery of physical activity programs in a semi-rural setting, offering critical insights into persistent barriers to meaningful participation. The study specifically aimed to determine student perceptions on enhancing the delivery of physical activity programs at a semi-rural university. Both the quantitative and qualitative findings highlight key gaps that hinder participation particularly issues related to facility upkeep, communication effectiveness, and institutional support for students.

The quantitative results demonstrated general agreement that the university's sports grounds and facilities were clearly marked and relatively well-maintained. However, some areas such as the condition of changing rooms and inadequate lighting received lower ratings. These findings are consistent with an earlier study conducted by Nxumalo and Beetge (4), which identified poor facility conditions and lack of safe access as key deterrents to participation among female students at the same institution. Similarly, Kgokong et al. (10) and Mthethwa (6), reported that deteriorating infrastructure discourages participation in physical activity among university students. Collectively, these findings indicate that infrastructural shortcomings continue to restrict engagement in semi-rural universities. Internationally, similar trends have been observed, with deteriorating facilities discouraging participation among university students (18).

In contrast, students expressed dissatisfaction with the communication practices of the institution. While some respondents felt informed about the benefits of participation, many noted the lack of transparency regarding departmental achievements, budgets, and future plans. The absence of timely and clear communication echoes findings by Peters (9), who reported that poor promotion and information gaps significantly limited student engagement in physical activity programs at universities. Nxumalo and Edwards (19) further noted that students were often unaware of available sporting opportunities, suggesting that orientation and outreach strategies were insufficient. The qualitative findings deepen the understanding of issues raised in the quantitative phase, particularly regarding perceived inequities in support and visibility support. A prominent theme was the lack of awareness and visibility of sport programs particularly among first-year students. Many participants only discovered available programs well into their academic careers, suggesting that enhanced orientation events, workshops, and increased social media use. Consistent with recommendations in the South African literature (6, 9, 19), students suggested improved orientation events, workshops, and increased use of social media to address this gap.

Support for student-athletes also emerged as a significant concern. Many felt that their sporting codes received unequal attention and resources, with female athletes particularly vocal about disparities in recognition and equipment. This perception of favouritism undermines student motivation and contradicts the ideals of equitable campus sport culture. Comparable observations have been reported in other local studies, where institutional support gaps affected student engagement and satisfaction (4, 5, 8, 20). While Van Zyl (20), studied elite student-athletes, our findings suggest that inequities in recognition and support are not confined to competitive athletes but affect all students engaging in physical activity in semi-rural universities. Participants also expressed that poorly trained or absent coaches diminished their sport experiences and discouraged long-term involvement. This aligns with earlier studies showing that quality coaching is essential for effective athlete development and sustained participation (8, 10). Financial constraints were also raised as a recurring issue, suggesting the need for subsidized programmes and bursary schemes to promote equitable access. Students described challenges of affording basic equipment, gym memberships, and travel-related costs. Suggestions included sports bursaries, discounted gym access, and formal recognition of achievements, recommendations that are consistent with findings from Nxumalo and Edwards (19), Nthangeni et al. (8). International comparisons also highlight similar patterns, where subsidized programs and scholarships increase engagement (21).

Facility related concerns extended beyond general maintenance. Poor lighting, unsafe playing surfaces, and shared facilities created logistical and safety challenges that discouraged consistent training. These concerns were corroborated by field observations conducted by the lead researcher, who noted overused fields, overcrowded indoor facilities, and a lack of essential resources such as lighting and nets. Although not the primary focus of this study, these observations reinforce student reports and support recommendations for more equitable and transparent resource allocation across all sporting codes to prevent bias and enhance accessibility. Similar findings were reported by Johannes et al. (3), who showed that students at a historically disadvantaged South African university faced comparable infrastructural and accessibility barriers, underscoring the persistent impact of resource constraints on student engagement.

Beyond institutional shortcomings, challenges such as lack of time, distance to facilities, and scheduling conflicts have also been cited in other university contexts. These findings resonate with South African evidence showing that semi-rural students often struggle to balance academic workload with participation in structured physical activity (7, 10). Van Zyl (20) highlighted that university student-athletes face challenges balancing dual careers due to institutional support gaps and limited recognition; our findings suggest that these support gaps extend to all students engaging in physical activity. International studies report analogous patterns, such as limited time and distant exercise venues (21, 22) and poorly scheduled facility hours (23), but the present study emphasizes the primacy of infrastructural and institutional barriers in this semi-rural context.

Overall, the findings underscore the interconnected nature of communication, infrastructure, staff engagement, and institutional support in shaping student participation. Addressing these challenges holistically through improved messaging, equitable resourcing, qualified personnel, and financial support could meaningfully enhance student engagement in sport and physical activity within semi-rural university contexts.

## 5 Strengths and limitations

The findings of this study may be restricted to other settings due to its focus on a single semi-rural university. Furthermore, the use of self-reported data may introduce bias, as participants may not always accurately report their perceptions or behaviours. In order to enhance the external validity of the findings, future research may consider the expansion of the sample to include multiple institutions. Subgroup analysis based on sport participation status was not conducted. Future studies should explore whether perceptions differ by engagement level, gender, or type of sport. Furthermore, the perspectives of sport administrators were not included, which limits understanding of the operational challenges, policy execution, and institutional dynamics influencing sport and recreation services. Lastly, this study did not assess students' understanding of budget allocations or their perceptions of equity in resource distribution. Future research could investigate financial governance structures and students' views on fairness in sport funding and resource access.

Despite these limitations, the study has notable strengths. It used a mixed-methods approach, combining quantitative breadth with qualitative depth to produce an understanding of students' perceptions. The inclusion of students holding leadership or committee roles in sport provided insight from individuals directly involved in campus sport structures. Furthermore, triangulation through field observations added contextual richness to the qualitative data. These strengths enhance the credibility and practical relevance of the findings, offering actionable insights for institutional policy reform in semi-rural higher education settings.

## 6 Conclusion

This study explored students' perceptions on enhancing the delivery of physical activity programs at a semi-rural university and gathered their recommendations for improvement. The findings reveal critical shortcomings in infrastructure, communication, and institutional support that hinder student engagement in physical activity. Students identified key areas needing reform, including clearer promotion of programmes, equitable resource allocation across sporting codes, access to qualified coaching staff, and formal recognition of athlete contributions. Beyond identifying these challenges, the study advances knowledge by centering student voices informing evidence-based improvements. The use of a mixed-methods approach provides both depth and breadth to the analysis, offering a well-rounded understanding of delivery gaps in under-resourced higher education settings.

To foster a supportive and inclusive environment for physical activity/sport participation, institutions should prioritise targeted institutional reforms such as improving facility maintenance, enhancing communication strategies, investing in staff training and engagement, and implementing structured support systems for student-athletes. These findings provide practical guidance for institutional policy reform and serve as a foundation for universities in similar semi-rural contexts seeking to enhance physical activity participation and student well-being.

## Data Availability

The raw data supporting the conclusions of this article will be made available by the authors, without undue reservation.
