# Supplementary material for: Student perceptions on enhancing the delivery of physical activity programs in a semi-rural university
Source: Front Sports Act Living. 2025 Oct 1;7:1627202. doi: 10.3389/fspor.2025.1627202 (PMC12521217; doi:10.3389/fspor.2025.1627202)
Supplement: Supplementary file 1 [file Supplementaryfile1.docx]

Table S1. Factor Loadings of students’ perceptions of the university’s sport and recreation services.

| My institution has…. | Factor | |
| --- | --- | --- |
|  | 1 | 2 |
| Communicated the achievements of the Sport Admin Department | .877 |  |
| Communicated the challenges faced by the Sport Admin Department in delivering sport services | .843 |  |
| Communicated the UNIZULU’s Sport and Recreation budget for the coming year | .842 |  |
| Communicated plans to improve service delivery in the coming year | .795 |  |
| Communicated the responsibilities of Sport Administration Department officials | .713 |  |
| Informed me about the benefits of participation in physical activities | .487 |  |
| Sport grounds facilities that are well maintained |  | .898 |
| Clearly marked grounds and sport facilities |  | .709 |
| Clean and well-maintained changing rooms |  | .557 |

Table S2. Summary of Perceptions Extracted Factors, Variance and Reliability statistics

| Factor | Construct | Items included | Variance extracted | Cronbach’s alpha |
| --- | --- | --- | --- | --- |
| 1 | Communication  (PER_COM) | - Communicated the achievements of the Sport Admin Department. - Communicated the challenges faced by the Sport Admin Department in delivering sport services. - Communicated the UNIZULU’s Sport and Recreation budget for the coming year. - Communicated plans to improve service delivery in the coming year. - Communicated the responsibilities of Sport Administration Department officials. - Informed me about the benefits of participation in physical activities. | 47.5 | .895 |
| 2 | Facilities (PER_FAC) | - Sport grounds facilities that are well maintained. - Clearly marked grounds and sport facilities. - Clean and well-maintained changing rooms. | 11.9 | .771 |

Table S3. Results of the one sample t-test for each perceptions’ construct.

| Perceptions of UNIZULU sport and recreation | n | Mean | Standard deviation | t | df | p-value |
| --- | --- | --- | --- | --- | --- | --- |
| Communication  (PER_COM) | 299 | 3.0281 | .85906 | .565 | 298 | .572 |
| Facilities (PER_FAC) | 300 | 3.2250 | 1.01586 | 3.836 | 299 | <.001 |
